# Analysis of Causal Relationships by Structural Equation Modeling to Determine the Factors Influencing Cognitive Function in Elderly People in Japan

**DOI:** 10.1371/journal.pone.0117554

**Published:** 2015-02-06

**Authors:** Daisuke Kimura, Ken Nakatani, Tokunori Takeda, Takashi Fujita, Nobuyuki Sunahara, Katsumi Inoue, Masako Notoya

**Affiliations:** 1 Division of Occupational Therapy, Faculty of Care and Rehabilitation, Seijoh University, Tokai, Aichi, Japan; 2 Division of Health Science, Graduate School of Medical Science, Kanazawa University, Tokai, Aichi, Japan; 3 Department of Rehabilitation Sciences, Faculty of Allied Health Sciences, Kansai University of Welfare Sciences, Kashiwara, Osaka, Japan; 4 School of Health Sciences, College of Medical, Pharmaceutical and Health Sciences, Kanazawa University, Kanazawa, Ishikawa, Japan; University Of São Paulo, BRAZIL

## Abstract

The purpose of this study is to identify a potentiality factor that is a preventive factor for decline in cognitive function. Additionally, this study pursues to clarify the causal relationship between the each potential factor and its influence on cognitive function. Subjects were 366 elderly community residents (mean age 73.7 ± 6.4, male 51, female 315) who participated in the Taketoyo Project from 2007 to 2011. Factor analysis was conducted to identify groupings within mental, social, life, physical and cognitive functions. In order to detect clusters of 14 variables, the item scores were subjected to confirmatory factor analysis. We performed Structural Equation Modeling analysis to calculate the standardization coefficient and correlation coefficient for every factor. The cause and effect hypothesis model was used to gather two intervention theory hypotheses for dementia prevention (direct effect, indirect effect) in one system. Finally, we performed another Structural Equation Modeling analysis to calculate the standardization of the cause and effect hypothesis model. Social participation was found to be activated by the improvement of four factors, and in turn, activated “*Social participation*” acted on cognitive function.

## Introduction

The lifestyle factors necessary to maintain “brain health” are classified into “mental activity”, “social actions”, and “physical activity” and are considered to be necessary to prevent dementia [1]. Several studies have suggested the protective effects of social, leisure, or physical activities in preventing dementia [2–7]. Complex factors that influence cognitive function directly in dementia preventive programs may also have an indirect effect. Therefore, it is often difficult to determine which factors influence dementia prevention directly and which factors have an indirect influence.

Complex activities (specifically, mental, social and physical activities) possess a dementia prevention effect that cannot be easily identified. Therefore, it is necessary to conduct multivariate analysis of many factors associated with dementia in order to determine how each factor affects cognitive function. However, previous studies have not used a method that analyzes multiple factors related to dementia simultaneously to explain the influence of each factor on cognitive function. If a causal relationship between cognitive function and a specific dementia prevention factor can be identified by such analysis, that factor can be considered important in explaining the protective efficacy of certain activities and in more thoroughly understanding dementia.

Dementia prevention interventions generally follow two strategies: a high-risk strategy for elderly people with a precursor stage of dementia onset (mild cognitive impairment, MCI) and a population-based strategy for all elderly people [8]. For the population-based strategy, there is no distinction between healthy people and people with MCI. Elderly people themselves seek to reduce their risk for dementia onset by participating in various activities [8]. In other words, in the population-based strategy, various activities (preventive factors) are related, and through a potentiality factor called “activity participation” these activities indirectly influence cognitive function as assumed based on the intervention theory hypothesis.

On the other hand, for the high-risk strategy, there is a focus on people with MCI. The medical professions, including occupational therapists, speech and language therapists and public health nurses, intervene directly after a diagnosis of MCI [8]. In other words, the causal structure is based on the theory that intervention directly influences cognitive function.

**Fig 1:**
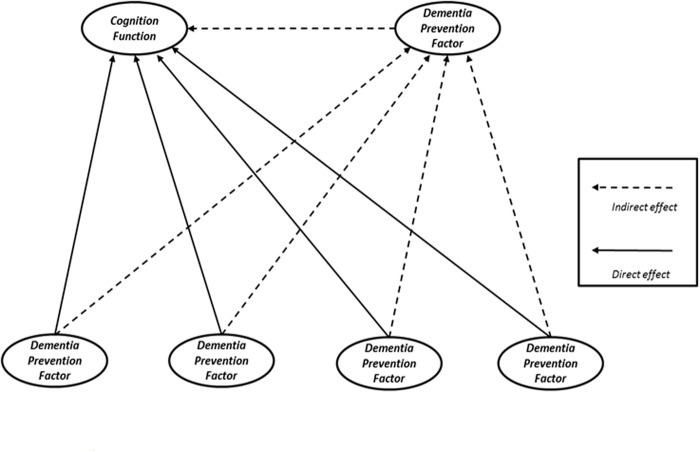
Cause and effect hypothesis model. Factors in the population-based strategy (direct factors) are shown in solid lines. Factors in the high-risk strategy (indirect factors) are shown in dotted lines.

Both of these intervention theories were recommended by the Ministry of Health, Labour and Welfare, which is the organization with jurisdiction over dementia prevention programs in Japan [8]. Consequently, some large-scale projects have investigated cognitive function and a prevention study has been conducted in order to show the effects of dementia prevention. As described below, the Taketoyo Project Aichi Prefecture in Japan the authors participate in is one of these projects. However, no previous studies have used a method for analyzing multiple factors related to dementia prevention simultaneously in order to explain the influence of specific factors on cognitive function. Therefore, the cause and effect model was selected as the model for the two intervention theory hypotheses that were incorporated into one system (Fig. 1).

The purpose of the present study was to identify effective preventive factors for cognitive function. In addition, this study aims to clarify causal relationships by determining what kind of process such a potential factor uses to influence cognitive function. First, each variable was categorized by factor analysis. Then, a model was constructed in which a category affected cognitive functional decline. Finally, a Structural Equation Modeling analysis was performed to calculate the standardization coefficient and correlation coefficient for every factor.

## Methods

### Subjects and procedures

The Taketoyo Project (TKP) is a community intervention research study being conducted in Taketoyo Town, Aichi Prefecture, based on a population-based strategy targeting elderly community residents. This research is based on the following concepts: 1) intervention in the social environment to address the needs of individuals is superior to a population-based strategy; 2) to allow citizens to participate easily, it is necessary to establish locations (salons) throughout the town instead of setting up only a few at the center of the town; 3) the large amount of manpower required to operate facilities in multiple locations can be covered by relying on volunteers instead of using professionals exclusively; 4) municipal support is essential; and 5) in addition to health exercises, a variety of fun programs should be offered. The intervention program aims to prolong the health of the elderly at the individual level and maintain a safe and comfortable community at the community level. In addition, the program seeks to prolong the healthy life years of the entire community by increasing physical activities and opportunities for going out, which increase citizens’ enjoyment in terms of mental cognition, improvement of physical/mental cognition through the enhancement of social support, and their advancement in social function within the community [9].

The subjects were the 366 elderly community residents who participated in TKP from 2007 to 2011. Thus, the analysis was performed on the totaled Year 1 data of 63 subjects participated from 2007, 75 subjects from 2008, 95 subjects from 2009, 65 subjects from 2010, and 68 subjects from 2011. The characteristics of the participants in the first year of the intervention are shown in Table 1. In TKP, an evaluation via the “OGENKI-check” (OGENKI means healthy in Japanese) is carried out once a year in September, starting in 2007. The “OGENKI-check” means healthy check in Japanese language. The “OGENKI-check” is comprised of a self-administered questionnaire, a test of cognitive function, and a test of physical strength and fitness. Among the “OGENKI-check” items used in this research, those questions corresponding to the following factors were selected as influencing factors for the prevention of cognitive function: mental, social, life, physical and cognitive functions, which were categorized as influencing factors for prevention of cognitive function in the review by Stuck et al. [10]. The self-administered questionnaires are comprised of basic attributes in health behavior, mentality, sociality and life function. As measures of health behavior, participants were asked about their smoking status and daily time spent for walking. For mentality, depression and self-efficacy were evaluated. For sociality, social network, social support, and communication were assessed. The answers given on the questionnaires are thought to indicate level of cognitive function. Accordingly, the Likert scale was used for part of the answers on the questionnaires as a means to remove the influence of cognitive function. For competence of life function, the Tokyo Metropolitan Institute of Gerontology Index of Competence (TMIG) was used. The Mini-Mental State Examination (MMSE) was used to evaluate cognitive function. The purpose of this study was not to investigate particular types of cognitive decline; rather, its aim was to evaluate many subjects simultaneously. Hence, the MMSE was used. As preceding studies have often used the MMSE in isolation when assessing a number of subjects, we also adopted the method in our study [11–12]. The elderly participants in the TKP participate in an annual “OGENKI-check”. The evaluations are administered by undergraduate students studying occupational therapy. Prior to the implementation of this evaluation, the students take two lectures on cognitive function evaluation and physical fitness measurement. Subsequently, the students are assessed by a faculty-conducted final check on their questioning technique and their knowledge of measurement/evaluation methods. Only students deemed capable of implementing evaluations were chosen as evaluators.

**Table 1:** Participant characteristics (n = 366).

Sex	Male：51 Female 315		
		means	SD
Age		73.7	6.4
Geriatric depression scale-15 (GDS15) (15point)		2.7	2.9
Instrumental activity of daily living(IADL) (5point)		4.8	0.5
Intellectual activities (IA) (4point)		3.5	0.8
Social Role (SR) (4point)		3.6	0.8
Serial 7 (5point)		2.9	1.7
3-word delayed recall(DR) (3point)		2.3	1.0
Word fluency(WF) (5point)		4.4	1.4
Grip strength(GS) (kg)		22.4	5.8
Chair stand test(CS)(times)		16.4	5.4
		Number	%
Self-rated health assessment (SHA) (5point)	excellent	46	12.6
	good	271	74.0
	average	35	9.6
	fair	11	3.0
	poor	3	0.8
Number of friends engaged in activities(NFEA)	excellent	63	17.2
(5point)	good	185	50.5
	average	107	29.2
	fair	7	1.9
	poor	4	1.1
Number of companions(NC) (5point)	excellent	23	6.3
	good	170	46.4
	average	148	40.4
	fair	22	6.0
	poor	3	0.8
15-minute walk(Walk) (5point)	Yes	313	85.5
	No	53	14.5
Frequency of going out(FGO) (5point)	every day	179	48.9
	2~3times/week	132	36.1
	1times/week	37	10.1
	1~2 times /month	16	4.4
	sometimes/year	2	0.5
	nothing	0	0.0

### Measures

The observed variables used to compose a potentiality factor of this study are shown in Table 2. For mentality, the self-rated health assessment (SHA) and Geriatric Depression Scale-15 (GDS) were used. For sociality, the frequency of going out (FGO), the number of companions (NC), and the number of friends engaged in activities (NFEA) were used. For life function, we used the following subordinate items of TMIG: instrumental activities of daily living (IADL), intellectual activity (IA), and social role (SR). For physical and cognitive factors, 15 minutes’ walk (WALK), grip strength (GS), chair stand (CS), serial 7s, 3-word-delayed-recall test (WDR), and word fluency (WF) were used.

**Table 2:** Main outcome measure.

***Physical factors:***
1) 15 minute walk (Walk)
2) Grip strength (GS)
3) Chair stand test (CS)
***Cognitive functions:***
1) Serial 7s
2) 3-word-delayed-recall (DR)
3) Word fluency (WF)
***Mental factors (Self-administered questionnaire):***
1) Self-rated health assessment (SHA)
2) Geriatric depression scale-15 (GDS15)
***Social factors (Self-administered questionnaire):***
1) Frequency of going out (FGO)
2) Number of companions (NC)
3) Number of friends engaged in activities (NFEA)
***Life function (Self-administered questionnaire):***
1) Instrumental activity of daily living (IADL),
2) Intellectual activities (IA)
3) Social role (SR)

### Statistical analysis

Statistical analysis was conducted according to the following three-stage approach. First, factor analysis was conducted to identify groupings within each category of mental, social, life, physical and cognitive functions. In order to detect clusters of 14 variables, the item scores were subjected to confirmatory factor analysis using the principal component method with varimax rotation. Factor analysis is a construct validity tool that aims to identify underlying clinical dimensions. The validity of a symptom cluster has been defined as the common variance of the factor and the construct validity is studied by comparison with other constructs [13]. Factors with an eigenvalue exceeding 1.0 and an interpretable constellation of items are usually considered of interest for the clinical description. Secondly, we constructed a model in which a category affected cognitive functional decline. Finally, we performed Structural Equation Modeling analysis to calculate the standardization coefficient and correlation coefficient for every factor. We assessed data model fitness with the Goodness of Fit Index (GFI), Adjusted Goodness of Fit Index (AGFI), and Root Mean Square Error of Approximation (RMSEA). The goodness-of-fit was evaluated by the following criteria: GFI >0.90, AGFI >0.90 [14], and RMSEA <0.05 [15]. Analyses were performed using Statistical Package for the Social Sciences (SPSS), version 18 (SPSS, Inc., Chicago, Illinois, USA) and SEM was performed using SPSS Amos 19.0 (SPSS, Inc.).

### Ethics Statement

In the Present study, prior to the implementation of the evaluation, a document explaining the purpose of the evaluation was distributed at each salon. The same document was also distributed on the day of implementation. After explanation, the participants who agreed to study entry did the signature of the agreement on the cover sheet of the questioner. With these procedures, as for the participants, informed consent was carefully confirmed. The study was conducted in accordance with the ethical standards set forth in the Helsinki Declaration (1983). The entire study protocol was approved by the Seijoh University Expert Committee on University Research Ethical Evaluation (“The studies on comprehensive support for the dementia prevention”, 2013C00015). And this study is in compliance with the research agreement established with Taketoyo Town.

## Results

### Result of Factor analysis

First, Kaiser-Meyer-Olkin (KMO) and Bartlett’s tests were performed to determine whether the factor analysis using 14 variables was suitable. The KMO test score was 0.70 and was judged to be a desirable value as a sample. In addition, the p-values of Bartlett’s test indicating the correlation between variables rejected a null hypothesis in p < 0.001. These results showed that the factor analysis using 14 variables was suitable. Therefore, the 14 variables scored were analyzed in factor analysis. Factor analysis of the 14 variables scored in the 366 participants resulted in several factors with eigenvalues exceeding 1.0. We will first present the five-factors solution (contribution 31.5%) (Table 3).

**Table 3:** Results of Factor analysis.

Factor						
	RL	CF	SS	IIADL	PF	communality
GDS	**-0.693**	-0.253	-0.032	-0.298	0.277	*0.498*
SR	**0.602**	0.189	0.039	0.417	-0.127	*0.400*
SHA	**-0.556**	-0.105	-0.039	-0.247	0.202	*0.315*
WF	0.213	**0.798**	0.15	0.272	-0.028	*0.645*
DR	0.204	**0.471**	0.085	0.13	-0.119	*0.233*
NC	-0.033	0.061	**0.516**	0.082	0.141	*0.337*
NFEA	-0.124	0.022	**0.424**	0.076	-0.022	*0.219*
CS	0.322	0.257	0.397	0.245	-0.35	*0.280*
IADL	**0.469**	0.219	0.284	**0.603**	-0.183	*0.410*
IA	0.363	0.049	0.099	**0.477**	-0.23	*0.261*
Serial	0.114	0.239	0.175	0.374	-0.192	*0.190*
FGO	-0.235	-0.009	-0.092	-0.288	**0.551**	*0.329*
GS	0.29	0.041	0.317	0.255	-0.330	*0.280*
Walk	-0.055	-0.045	0.067	-0.012	0.264	*0.093*
*Contribution*	*2.101*	*0.842*	*0.729*	*0.458*	*0.281*	*4.411*
*%*	*15*	*6*	*5.3*	*3.2*	*2*	*31.5*
Factor correlation matrix contains						
Factor	RL	CF	SS	IIADL	PF	
RL	1.000	0.288	0.502	0.063	0.410	
CF	0288	1.000	0.298	0.185	0.137	
SS	0.502	0.298	1.000	0.314	0.415	
IIADL	0.063	0.185	0.314	1.000	0.204	
PF	0.410	0.137	0.415	0.204	1.000	
Kayser-Meyer-Olkin measure of sampling adequacy					0.726	
Bartlett’s test			p valule		0.000	

Bold indicates factor loading ≥ 0.4.

Role in life (RL), Cognitive function (CF), Social skill (SS), Intellectual IADL (IIADL), Physical function (PF), Self-rated health assessment(SHA), Geriatric Depression Scale-15 (GDS15), frequency of going out (FGO), the number of companions (NC), the number of friends engaged in activities (NFEA), Instrumental Activity of Daily Living (IADL), Intellectual activities (IA), Social Role (SR), 15 minute walk (Walk), Grip strength (GS), Chair stand (CS), Serial 7s, 3-word-delayed-recall test (DR), Word fluency(WF)

The first factor shows the factor loading that is high for four items of “Self-rated health (SH)”, “Geriatric Depression Scale-15 (GDS)”, “Social role (SR)”, and “IADL” (absolute value of the factor loading ≥0.4). “Social role (SR)” and “IADL” are items that indicate a “Role in life”. “Self-rated health (SH)”, and “Geriatric Depression Scale-15 (GDS)” are mental factors which may become the base of “role in life.” Thus, in several aspects, factor 1 agrees with “role in life”. Factor 2 shows a factor loading that is high for the two items “3-word-delayed-recall test (WDR)”, and “Word fluency (WF)”. Thus, the structure of the second factor agrees with “cognitive function”. Factor 3 shows a factor loading that is high for the two items, “the number of companions (NC)” and “the number of friends engaged in activities (NFEA).” It is understood that the presence of many friends reflects “Social skill.” Thus the structure of the third factor agrees with “Social skill”. The structure of factor 4 agrees with the categorization of “Intellectual IADL”, including the two items “instrumental activities of daily living (IADL)” and “intellectual activity (IA)”. Factor 5 included one item, “frequency of going out”, and the structure of the fifth factor is thought to represent “physical function”.

### Result of Structural Equation Modeling analysis

Factor analysis of the 14 variables scored in the 366 participants showed a five-factor solution. Therefore, these factors were used to construct a model in which a category affected cognitive functional decline. We analyzed this model using SEM.

**Fig 2:**
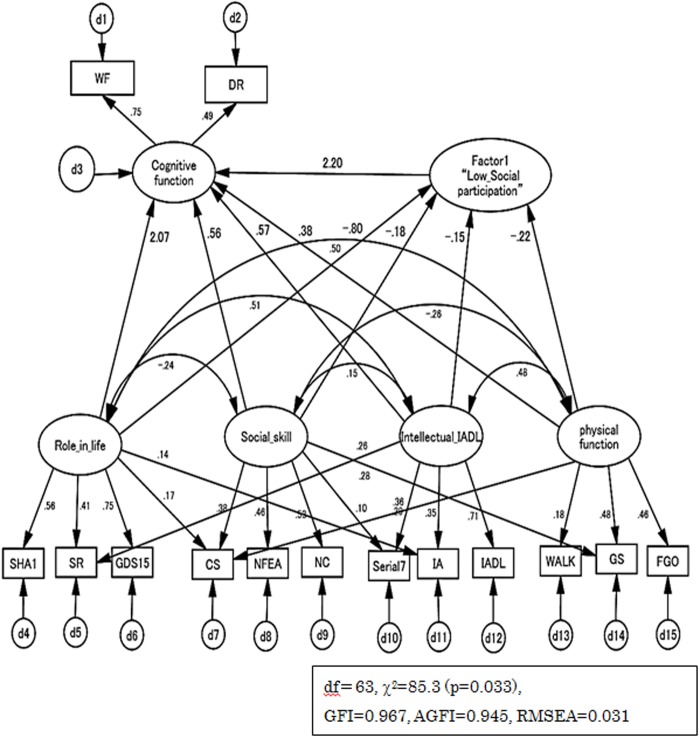
Results of Structural Equation Modeling. GFI = Goodness of Fit Index, AGFI = Adjusted Goodness of Fit Index, RMSEA = Root Mean Square Error of Approximation.

The p-value for the model fit chi-square (79.9, DF = 61) was 0.053, and GFI, AGFI and RMSEA were 0.970, 0.948, and 0.029, respectively. Thus, all three fitness statistics indicated an excellent fit to the overall model. The final estimated model, with standardized path coefficients, is presented in Fig. 2.

In interpreting the analysis, Factor 1 received influence from “Role in life”, “Social skill”, “Intellectual IADL” and “Physical function”, giving a negative standardization coefficient from four potentiality factors but a negative standardization coefficient for “cognitive function”. Therefore, it was understood that Factor 1 was a positive factor and was named “Low Social participation”.

The “Cognitive function” indicator variables (direct effect), “Role in life” (path coefficient = 2.07), “Social skill” (path coefficient = 0.56), “Intellectual IADL” (path coefficient = 0.57), “Physical function” (path coefficient = 0.38), and “F1: Low Social participation” (path coefficient = 2.20) loaded onto the “Cognitive function” factor. The “F1: Low Social participation” indicator variables (direct effect), “Role in life” (path coefficient = -0.80), “Social skill” (path coefficient = -0.18), “Intellectual IADL” (path coefficient = -0.15), and “Physical function” (path coefficient = -0.22), loaded onto the “F1: Low Social participation” factor. On the other hand, the indirect effect indicator variables for “Cognitive function” through “F1: Low Social participation”, “Role in life” (path coefficient = -1.76), “Social skill” (path coefficient = -0.39), “Intellectual IADL” (path coefficient = -0.33), and “Physical function” (path coefficient = -0.48), loaded onto the “Cognitive function” factor.

Table 4 shows the comparisons between direct and indirect effects and the ratio among total effects. When we compared indirect effects with direct effects, “Role in life”, “Social skill”, and “Intellectual IADL” were high in direct effects, and “Physical function” showed a high indirect effect. However, after standardization of the direct effect and indirect effect coefficients, the ratio among standardization synthesis effects was up to 63%. In the present study, it is understood that there is not a difference when the ratio among total effects is about half, but that there is a difference when the ratio is around 90% [16]. Therefore, from these findings, it was interpreted that both direct and indirect effects influenced cognition function.

**Table 4:** Direct and indirect coefficients.

Standardized coefficients	RL	SS	IIADL	PF
Direct effect	**2.07(54.0%)**	**0.56(58.9%)**	**0.57(63.3%)**	0.38(44.1%)
Indirect effect	-1.76(46.0%)	-0.39(41.1%)	-0.33(36.7%)	**-0.48(55.9%)**
Total effect	3.83(100.0%)	0.95(100.0%)	0.90(100.0%)	0.86(100.0%)

Bold font indicates high values of standardization coefficients.

Role in life (RL), Cognitive function (CF), Social skill (SS), Intellectual IADL (IIADL), Physical function (PF)

The general concept of Low Social Participation is written in our description in italic font (*Social participation*) to distinguish it from the potentiality factor.

Four factors of “Role in life”, “Social skill”, “Intellectual IADL”, and “Physical function” have direct effects on the maintenance of “Cognitive function”. In addition, improvements in these four factors activate “Social participation.” Activated “Social participation” acted as a direct effect on cognitive function (see upper right of Fig. 1.)

## Discussion

In this study, a hypothetical model of cause and effect, which schematizes preventive factors for dementia, was developed. The process of dementia prevention was analyzed in participants in a dementia prevention program based on a population-based strategy. “Physical function” was shown to be directly involved in cognitive function, as were “Role in life”, “Social skills”, and “Intellectual IADL”, with a reciprocal relationship among them. Furthermore, it was successfully demonstrated that these preventive factors for dementia are also indirectly involved in the cognitive function mediated by the factor of *Social participation*, for the first time.

“Role in life”, “Social skills”, “Intellectual IADL”, and “Physical function”, which are shown in this study, as well as *Social participation*, which is an indirect factor, are life habits (behaviors) of the elderly cultivated through their social life experiences in the past. The effects of lifestyle on development of diseases are cumulative in both positive effects and negative effects as typified by Brinkman index [17] for smoking. Daily habits such as “Roles in life”, “Social skills” and “Intellectual IADL” are established by the middle-age period in the human life. It can be presumed that the influence from such daily habits should last through the senior years.

“Roles in life” are parts of life, and it is considered that it is important to fulfill some roles (for example, taking the grandchildren to and from nursery and school, and preparing meals) regardless of the size of the family household. That means it is actually not good for the elderly if the younger generation does everything instead of letting elderly adults play a role in the family life. Meanwhile, “Social skills” basically contribute to building the relationships among people including making friends. It is considered that such “Social skills” are established based on the formation of an interpersonal relationship during the developmental stage in childhood and are also influenced by their experience of how much and what kind of roles the person has played, for example, roles at parents’ meeting of nursery, kindergarten and schools, and roles in the neighborhood and the local community. “Intellectual IADL” is assumed to include actions such as setting up a bank account and making a deposit, using a cash card, and getting a mortgage to buy a house. For “Physical functions”, there are already many previous research studies that have identified “Physical function” as a preventive factor [18–20]. Perhaps the focus on “Physical function” is because it is relatively easy to promote functional alteration of muscles and the nervous system even in older age.

As explained above, it can be interpreted that these preventive factors determined in this study are caused by daily habits (behaviors) before the older age. Preventive measures for dementia may need to be aimed at younger and midlife adults in the future. In other words, according to the results obtained in this study, it is presumed that few behaviors are formed later in life. For instance, “Roles in life” are related to whether basic daily habits (household chores such as cleaning, laundry and cooking) are acquired by helping with the housework during childhood and adolescence, as well as performing a job in an organization such as a company after being employed. Then, such behaviors from when they were actively working influenced the behaviors of the elderly. Therefore, an overall view of the entire life history of the person may be required to understand the effects on dementia prevention.

According to Erikson, success or failure in solving issues at each stage of development greatly influences the subsequent developmental stages, and hence intervention in or prior to middle age seems necessary in order to assist people in passing through developmental issues [21].

On the other hand, it has been reported that at least ten years before the onset of symptoms of cognitive impairment (particularly Alzheimer’s) there is already accumulation of Aβ, tau proteins, and other abnormal proteins [22]. Moreover, and though limited to cases of genetically acquired Alzheimer’s, the international study of Alzheimer-type cognitive impairment—Dominantly Inherited Alzheimer Network (DIAN)[23]—has shown that the accumulation of Aβ in the brain can be found 20 or more years before the onset of Alzheimer’s symptoms. Thus, biomarkers also show that abnormal accumulation of proteins in the brain begins some time before the onset of cognitive impairment, suggesting that it is necessary to intervene in some way before old age begins.

The population-based strategy is an interventional strategy that aims to maintain and improve cognitive functions through social participation. Some previous studies have concluded that social participation is related to changes in cognitive functions [24–25] and has protective effects on cognitive functions. In addition, although this study identified relationships with the cognitive functions of four preventive factors for dementia, which have already been suggested in previous studies [26–30], this study newly revealed that four preventive factors for dementia not only directly influence the cognitive functions without being mediated by *Social participation*, but also indirectly influence the functions through *Social participation*. In other words, four factors, “Physical function” in addition to “Roles in life”, “Social skills” and “Intellectual IADL”, establish a strong foundation for cognitive functions. These factors are also important as they support the factor of *Social participation*. The fact that “Physical function” becomes the foundation to support *Social participation* and enriches *Social participation* in addition to “Roles in life”, “Social skills” and “Intellectual IADL” indicates that these factors have a positive relationship with the cognitive function.

In the high-risk strategy, which has an interventional strategy considering preventive factors for dementia as direct factors in maintaining cognitive function, it is considered that the direct involvement of experts with advanced techniques is effective for preventing dementia [8]. To support and maintain factors of “Physical function” in addition to “Roles in life”, “Social skills”, and “Intellectual IADL”, it is important to extend this to the population-based strategy and who can develop suitable interventional methods.

The provision of opportunities for *Social participation* is actively promoted in Japan. *Social participation* is regarded as an important factor contributing to creating a healthy and purposeful life for the elderly. However, considerable manpower is required to organize *Social participation* in many locations, and human resources are limited to the placement of experts. Effective utilization of social resources such as volunteers is required to compensate the limitation. Recently, it was reported that local communities could gain numerous benefits through involvement of the elderly with a great deal of social skills in a regional contribution [31]. Not just participation in social activities, but also motivation for the regional contribution is sought for the elderly in a rapidly aging society.

As mentioned above, manpower needs can be met through the participation of elderly volunteers in activities, but the absence of an expert viewpoint is a problem in the population-based strategy. In order to address this problem, efforts to involve experts in educating volunteers and others are beginning to be conducted [32]. A business operation facilitating the use of volunteers has been launched, and occupational therapists are engaged in a training program for involving volunteers in the TKP [33]. In addition, it has been reported that improvement of cognitive function was achieved by implementing an interventional program that was designed and supervised by experts [27]. Therefore, as demonstrated by the results of the present research, if the population-based strategy has indirect effects in addition to direct effects, the involvement of experts is required for cultivation of leaders and volunteers with knowledge and skills based on a scientific foundation.

Development of a dementia prevention program based on a population-based strategy that includes both direct and indirect interventional effects is practical. However, it is desirable to provide opportunities to promote behavior change starting from middle age, the prime working years, to encourage the elderly to contribute such activities.

Based on these considerations, it is considered that the interventional methods and involvement of experts who can evaluate the effect of the intervention for the factors including “Roles in life”, “Social skills”, “Intellectual IADL” and “Physical function”, which are obtained in this study, are required in the dementia prevention program based on the population strategy. In addition, it is also important to encourage changes in behaviors of the actively working generation even before middle age. Accordingly, future research will have to accumulate study results from the beginning of interventions.

Finally, we will add an explanation regarding cognitive function assessment and differences by sex. Preceding studies indicate that past experiences affect current social participation in both men and women [34–35]. Although men and women do have different lifestyles, it seems that the important factor in present social participation is past experiences of social participation, not the sex of the subject. Accordingly, although most of the subjects in this study were women, our study did not subdivide subjects by sex. The other hand, assessment of cognitive function in this study was performed using MMSE in isolation, as described in the Procedures section. This measure was taken as it was necessary to assess many subjects simultaneously in a short time, despite there being limitations to its interpretation.

### Limitations

The present study has several limitations. First, the subjects are dementia prevention service participants in a single area. Therefore, the perspective among community-dwelling elderly may not be assessed. Intelligence and the analysis in many areas are necessary for the generalization of the result. Second, the distribution of men and women in this study subject may differ from the distribution of sex in other dementia prevention programs. It is difficult to control for sex in the current analysis. Despite these limitations, a causal relationship between cognitive function and dementia prevention factors was shown by analysis of many factors simultaneously in the present study. It is thought that it is important to foster social participation and contribution activity of the future elderly people to improve dementia prevention.

## Conclusion

The purposes of this study were to identify potential factors that affect decline in cognitive function and clarify causal relationships. Social participation is activated by the improvement of the four identified factors, and the activated Social participation acted directly on cognitive function.
